# Education Program for Enhancing Health Care Students’ Attitudes Toward People Living With Dementia: Protocol for a Single-Arm Pre-Post Study

**DOI:** 10.2196/62654

**Published:** 2024-09-18

**Authors:** Dianis Wulan Sari, Haruna Kugai, Ayumi Igarashi, Manami Takaoka, Hiroshige Matsumoto, Haruno Suzuki, Jinyan Wu, Rizki Fitryasari, Ike Ayunda Nasifah, Eka Mishbahatul M Has, Noriko Yamamoto-Mitani

**Affiliations:** 1 Faculty of Nursing Universitas Airlangga Surabaya Indonesia; 2 Department of Gerontological Home Care and Long-term Care Nursing The University of Tokyo Tokyo Japan; 3 Dementia and Aging Care Research Centre Universitas Airlangga Surabaya Indonesia; 4 Global Nursing Research Centre, Graduate School of Medicine The University of Tokyo Tokyo Japan; 5 Department of Community Health Nursing Division of Health Sciences & Nursing, Graduate School of Medicine The University of Tokyo Tokyo Japan; 6 Department of Physiological Nursing, School of Nursing University of California San Francisco, CA United States

**Keywords:** ageism, dementia, health professional, education program, long-term care

## Abstract

**Background:**

Health care students are instrumental in shaping the future of dementia care. Cultivating a positive attitude and understanding toward people living with dementia is crucial for diminishing the stigma associated with the condition, providing effective and person-centered care, and enhancing the quality of life for people living with dementia. Educational programs about dementia are increasingly recognizing the potential of gaming tools.

**Objective:**

This study aimed to evaluate the effectiveness of gaming-based dementia educational programs in improving attitudes toward people living with dementia among health care students.

**Methods:**

This single-arm pre-post study will be conducted among health care students in Indonesian universities. This educational program based on gaming tools will consist of a lecture on dementia, the use of N-impro (gaming tool), and the enactment of short dramas depicting desirable and undesirable communication with people living with dementia behaviors. We will assess attitudes toward people living with dementia, intention to help people living with dementia, knowledge of dementia, and the stigma associated with people living with dementia. The gaming-based dementia education program will be integrated into the curriculum of the health care students. The program will be implemented once with a duration of 90 minutes.

**Results:**

Data collection will occur from August 2023 to March 2024. Analysis of the data will be finalized by May 2024, and the outcome will be determined by July 2024. The impact of the gaming-based dementia educational program on improving attitudes toward people living with dementia will be reported. The study findings will be published in a peer-reviewed journal.

**Conclusions:**

The gaming education program demonstrates significant potential in enhancing attitudes toward people living with dementia across various countries, introducing an innovative method for the community-based support of people living with dementia.

**Trial Registration:**

ClinicalTrials.gov NCT06122623; https://clinicaltrials.gov/study/NCT06122623

**International Registered Report Identifier (IRRID):**

DERR1-10.2196/62654

## Introduction

The global increase in the aging population has increased the number of people living with dementia [1]. In Indonesia, the adjusted weighted people living with dementia rate is 27.9%, indicating a potential population of over 4.2 million people living with dementia [2]. Consequently, global policies have been established to foster a “dementia-friendly” society [3,4]. Dementia-friendly initiatives aim to enhance the quality of care for patients with dementia [5]. Distinguishing ageism from carefully reasoned clinical decisions can be challenging [6]. Health care professionals such as medical doctors, public health workers, nurses, dentists, pharmacists, and nutritionists may inadvertently perpetuate ageism, as it can be deeply embedded in daily procedures. As a result, older adults may not receive the same level of care as younger patients [7]. Therefore, health care professionals must improve their ability to provide dementia care to address the diverse needs of older adults and combat ageism.

A previous study found that medical students had neutral attitudes toward older individuals and lacked knowledge about dementia [8]. Whereas, a study in Indonesia found low knowledge but high attitudes toward dementia among nursing students [9]. Bachelor’s degree courses are offered to help health care professionals understand health care subjects, including holistic care for older adults with dementia. Health care students may struggle to care for older adults with dementia due to a lack of understanding of how to respond to specific symptoms of dementia and challenges in visualizing actual care scenarios [10]. Thus, it is necessary to pursue effective educational methods that enhance knowledge and attitudes toward older adults.

Health care professionals can provide quality care by combining integrated learning, critical thinking, and optimal decision-making skills. The integration of simulation into the educational process facilitates the attainment of this goal. A previous study has suggested that simulated education is beneficial for content that is challenging to convey through traditional classroom instruction [11]. One such method of simulation education is a gaming-based education program, which allows students to engage with and enjoy the learning process [12,13]. The implementation of dementia education through a game- and simulation-based program has been shown to effectively enhance knowledge and attitude toward dementia among health care students [14-17]. Therefore, an educational approach that introduces dilemmas through gaming, is needed [18].

In recent years, we have developed a dementia gaming-education program known as N-impro [19,20]. This program is designed to influence attitudes toward supporting older adults and foster community networks that promote an age-friendly environment within Japanese communities. Given the rapidly increasing demand for health care professionals and long-term care facilities to care for the older adults in Indonesia [21], the N-impro program will be adapted for health care students in Indonesia with necessary modifications to improve attitudes toward supporting older adults. Therefore, this study aims to assess the effectiveness of gaming-based dementia educational programs in enhancing health care students’ attitudes toward people living with dementia.

## Methods

### Study Design

This study will use a single-arm pre-post design to assess the effectiveness of gaming-based dementia educational programs for health care students. The study will be conducted in collaboration with the Faculty of Health at an Indonesian University. The programs will be integrated into courses related to dementia care, such as the Gerontological Nursing Care course, the Psychiatric Nursing course, and the Health Assessment course. Participation in the study is open to all students in these classes. However, there will be no penalties for students who choose not to participate. The study will continue until participant inclusion is completed, which is anticipated to be around March 2024.

### Participants and Settings

Participants in this study will be health care students at an Indonesian University. For this study, we will invite nursing and public health students. A participant must meet the following inclusion criteria: they must be a health care student enrolled in the semester for which we have designated the course for the implementation of this educational program. For instance, nursing students at Airlangga University will be selected from those in the fifth semester. While public health students are in the sixth semester, both are third-year grade health care students with class topics related to dementia. Prospective participants will be assigned a new ID number to ensure their student ID numbers remain confidential. The sample size was calculated using a power analysis for differences between two dependent means (matched pairs). The total sample size is 68 health care students with an effect size of *d*=0.5 (Reference from N-impro study: *d*=0.5, α=0.05, power=0.80). In conducting a matched pair *t* test analysis, this study will collect sufficient data from the participants; the sample size will exceed the calculated sample size.

This study will be conducted at Indonesian universities with health-based faculties. Health education activities will be implemented in various classrooms within the university. Each classroom will accommodate approximately 50 participants and will be equipped with chairs and tables to facilitate discussion. The classrooms will be divided into 4 parts, each comprising up to 10 chairs.

### Recruitment

Prospective participants will be identified using information from the e-learning portal of the university. We will document the data of the students enrolled in the designated course for the execution of this educational program at the start of the odd semester for the 2023-2024 academic year. Individuals who meet the eligibility criteria will receive detailed communications about the study and invitations to participate in the program.

The course coordinator and principal investigator (PI) will comprehensively explain the learning plan or syllabus for the designated course at the beginning of the semester. Generally, an overview of the execution of the research session will be presented, and detailed research procedures will be presented shortly before the research begins. An announcement regarding the research implementation will be distributed approximately 1 week in advance, and the recruitment process will be conducted through a Google form. Following the explanation of the research procedure, potential participants can freely choose whether or not to participate in the research. Those who express interest will receive a new ID for this study and will be notified of the schedule and location of the educational program through a class announcement 3 days before it commences.

On the event day, prospective participants will gather at the designated venue and formally record their attendance by signing an attendance sheet with their newly assigned ID. The PI overseeing this research will clarify all study phases, highlighting the participants’ right to withdraw at any point. Participation in this research will be entirely voluntary. Students who choose not to participate will continue to receive standard dementia-related materials through the e-learning portal of the university, with the exception of the game-based education program. All participants who consent to participate will express informed consent at the beginning of the Google form. Furthermore, we will state that we would regard the completion and submission of the questionnaires as consent to participate.

### Implementation

The intervention of this study is a gaming education program, which will be conducted once for each participant. The program will be implemented at various times, with each session accommodating a maximum of 60 participants. These participants will be divided into small groups of approximately 7-8 individuals within the same room, each group led by a facilitator. The facilitators, who will be either research team members or health lecturers, will be recruited and trained jointly by the Japanese and Indonesian research teams. The program’s instructors include the PI of this study, assisted by a research team serving as co-instructors.

#### Development of Gaming Education Program

The gaming education program was developed based on previous study [19,20,22]. The authors modified the study outline based on the participants and purpose of this study. The gaming educational program in this study comprises 3 primary components, as outlined in Table 1. The sequence of the short film (undesirable communication with people living with dementia), lecture, N-impro gaming, and the desirable communication with people living with dementia scenarios is determined based on insights from previous studies. The gaming education program is designed to be conducted within 90 minutes. The 3 primary components of the gaming education program are as follows.

**Table 1:** Overview of the gaming educational program.

The content of the education program			Time
**Introduction of the study**			
	Principal investigator explained the overall of study and content of inform	5 min	
**Pretest**			
	Participants will receive a QR code providing access to information regarding Informed Consent and the online questionnaire.	10 min	
**Short film (Undesirable communication with people living with dementia scenario)**			
	Observing a narrative featuring a woman people living with dementia, participants reflect on her background and the reactions of those around her.	10 min	
**Lecture**			
	The lecture consists of dementia, treatment of people living with dementia, person-centered care, and social support. The lecture included questions and answers.	25 min	
**N-impro gaming**			
	In the gaming setting, students will participate as a group alongside a facilitator. The game is structured with components such as “situation cards,” “answer cards,” and “point cards.” All groups will be provided with identical sets of situation cards.	20 min	
**Short film (Desirable communication with people living with dementia scenario)**			
	Participants identify differences in the emotional experiences of people living with dementia and the reactions of individuals in their immediate surroundings between the two given scenarios.	10 min	
**Posttest**			
	Participants will be issued a QR code to access the online posttest. In addition, there will be a designated space for participants to provide free-form comments expressing their opinions and feedback on the gaming education program.	10 min	

#### Short Drama Portraying Undesirable and Desirable Scenarios

Participants will view a short drama depicting an older woman who may be experiencing dementia. The narrative explores her life history, values, and interactions with family, friends, and community members. In total, 2 parallel scenarios are presented, showcasing both desirable and undesirable communication methods. This contrast enables the participants to subjectively experience the emotional responses of the main character to different types of communication; thus, the participants can naturally learn the appropriate method of communicating with people living with dementia. The scenario of the short drama was created based on interviews with people living with dementia and their families, a review of scholarly articles, and consultations with experts in dementia care. The research team translated a Japanese drama into Indonesian and added subtitles. The drama was projected onto a screen, akin to the setting of a movie theater.

#### A Lecture About Dementia

Lectures form the foundational framework of this educational program, which is based on the established curriculum for dementia-related health education. Furthermore, the contents of these lectures have been fashioned from the condensed dementia supporter training program in Japan [23,24]. These include the definition of dementia, diagnostic procedures, the various types of dementia, underlying causes, symptoms, treatment and prevention strategies, person-centered care and social support for people living with dementia, and the point of nursing care and how to support the people living with dementia.

Japanese researchers will curate the content outline, followed by their Indonesian counterparts, who will generate the presentation slides and conduct the lectures. A proficient senior lecturer will deliver the courses. The lessons will be systematically recorded and subsequently projected onto screens during class sessions to ensure uniformity across all instructional content in the various class settings.

#### N-Impro (Gaming Tool)

We developed the gaming tool based on the methodology of a disaster education program [25] and modified it to discuss dilemmas that can occur in a convenience store [19]. The game comprises “situation cards,” “answer cards,” and “point cards.” The situation cards present a 3-part scenario: (1) assigning a role to the participant (eg, you are a convenience store manager), (2) describing a relevant dilemma, and (3) prompting the participant to choose a course of action (yes or no). We developed eleven situation cards, each depicting a unique dilemma based on episodes from the interviews. For instance, the following situation card was created:

You are a convenience store manager. You suspect a customer has dementia and are concerned for his well-being. You have previously consulted a welfare volunteer about the customer; however, she said she can do nothing until the name or address of the customer is identified. The customer visits your store irregularly, and you are unable to learn his name because of his unintelligible.

Other situation cards were similarly crafted to reflect various dilemmas. The N-impro cards are available in Japanese and English. The professional translator translated into Indonesian (Multimedia Appendix 1).

The N-impro game will be played for about 25 minutes during the third session. The research team will provide 6-7 cards with the same scenario and order for each group. Each N-impro game group will consist of 7-8 students with 1 facilitator. The facilitators are teachers in the faculty of health care. The facilitator has undergone 2 training sessions. In the first session, training was conducted by a Japanese team, which included video demonstrations of the N-impro game and slides explaining the game rules. Then, in the second session, the research PI in Indonesia completed offline training for all facilitators to present the game rules and further discuss and role play the N-impro game.

### Data Collection

Data will be collected from November 2023 to March 2024 through an online questionnaire. Participants will be informed to prepare a mobile phone or personal computer before joining the class. For the online questionnaire, participants need to access the form, enter the required information, complete the questionnaire, and submit the form. Participants do not need to log into their Google accounts to access the link. They will only provide the new ID number at the beginning of the form. The link to the questionnaire will be shared through a QR code displayed on the classroom screen.

### Measurement

This study assesses the pre- and posttest outcomes of a gaming-education program focused on dementia. We aim to measure the effectiveness of the education program by evaluating attitudes toward people living with dementia, intentions to assist people living with dementia, knowledge of dementia, and stigma associated with people living with dementia. This study hypothesizes that a gaming-based education program will foster positive attitudes toward people living with dementia and an intention to help people living with dementia. Furthermore, we anticipate that the knowledge gained from the lectures and discussions during the gaming education will enhance the confidence of health care students in caring for people living with dementia.

This study uses the model by Lane and Yu, which encompasses 3 primary concepts contributing to dementia-friendly communities: knowledge of dementia, attitudes toward people living with dementia, and the intention to assist people living with dementia [26]. The model is underpinned by 2 theories: first, an increase in dementia knowledge is directly linked to a high intention to help people living with dementia, and second, attitude is an intermediary factor between knowledge and intention, influencing the formation of intention and potentially enhancing tangible supportive actions. Therefore, according to this model, improvements in both knowledge and attitude should strengthen the intention to assist.

The questionnaire used in this research is in Indonesian (Bahasa). The questionnaire was originally in English and Japanese. We performed a forward and backward translation into Indonesian using the standard method [27]. The translation process involves 2 stages. First, a health care professional who is fluent in both Japanese and Indonesian, has expertise in dementia, and possesses knowledge of Japanese and Indonesian culture performs an initial forward translation. Second, a professional translator who is proficient in Japanese-Indonesian language performs the back translation. Afterward, the Japanese research team validated the translations. Subsequently, we sought input from an Indonesian lecturer, an associate professor in the Department of Japanese Studies, Department of Gerontological Home Care and Long-term Care Nursing, University of Tokyo to ensure the fluency and uniqueness of sentences in Indonesian. The following are the measurements.

#### Primary Outcomes: Attitudes Toward People Living With Dementia

The primary focus of this study is the attitude toward people living with dementia, as it is intrinsically linked to the concept of attitude. The main objective of education aimed at fostering a dementia-friendly community is to enhance attitude. Attitude, as a concept, is a mental and neural state molded by experience, which influences the responses of individuals to various objects and situations and exerts a directive or dynamic impact on their behavior [28,29]. Consequently, numerous previous studies have used attitude as the primary outcome in educational research [22,30-32].

People’s perceptions of people living with dementia are related to stigma. Stigma is a societal phenomenon characterized by others perceiving an individual as deviating from the norm [33]. There are significant obstacles to creating communities that are supportive of people living with dementia, including stigma and social isolation. Stigma can impede the provision of care and support, frequently manifesting in behaviors such as excluding people living with dementia from health care [34,35]. Stigma can significantly influence the attitudes and behaviors of health care students. Therefore, measuring attitudes toward people living with dementia is important among health care services and students, as it relates to people’s perceptions of people living with dementia.

Attitudes toward people living with dementia were assessed using a 14-item measurement tool developed using information from previous research. This tool uses a 4-point Likert scale, ranging from 1 (disagree) to 4 (agree), and comprises 4 subscales: tolerance (5 items), refusal (4 items), feeling of distance (3 items), and affinity (2 items). The total score ranged from 14 to 56, with high scores indicating favorable attitudes. Tolerance was assessed with the statement, “I am open to having more interactions with people living with dementia in my daily life.” Refusal was measured by “I prefer to minimize interactions with people living with dementia as much as possible.” The feeling of distance was gauged with “If a family member were to develop dementia, I would be concerned about what people around me would think.” Affinity was evaluated by “If someone with dementia requires assistance, I would readily offer help.”

#### Secondary Outcomes

##### Knowledge of Dementia

Knowledge of dementia will be measured using the 15-item Dementia-Related Knowledge Scale developed by Kim and Kuroda [36], which assesses the knowledge of dementia: its symptoms, treatments, and diagnosis. This scale will require respondents to provide answers using a 3-point Likert scale ranging from agree, disagree, and don’t know. The author will calculate the total dementia knowledge score, with a high score indicating a high level of knowledge about dementia.

##### Ageism Among People Living With Dementia

Ageism is stereotyping, prejudice, and discrimination against individuals based on their age [37]. Dementia is often associated with advanced age. To develop dementia-friendly services by health care professionals, it is crucial to investigate the prevalence of ageism among people living with dementia. Thus, we will use the Fraboni’s Scale of Ageism (FSA) short version to assess the level of ageism among people living with dementia [38,39]. The FSA short version comprises 19 items that measure negative opinions toward older people using a Likert scale (1=strongly disagree, 2=disagree, 3=agree, and 4=strongly agree). Items that represent positive opinions toward older adults have reversed scoring (items 13-16). The possible FSA scores ranged from 19 to 76, with the high scores indicating high levels of ageism.

##### Intention for Helping Behaviors Toward People Living With Dementia

Based on previous research, methods aimed at raising awareness about dementia and providing education to individuals can enhance the probability of individuals engaging in supportive actions through 2 mechanisms. Individuals tend to develop favorable attitudes toward people living with dementia, and they improve their capacity to recognize signs indicating a need for assistance [40]. Therefore, the intention to help will be measured using 4 vignettes derived from the previous study. The vignettes describe situations in which people living with dementia may require assistance. For example, consider a scenario where you are a clerk in a supermarket. An older woman, a regular customer, arrived this morning to buy 2 bunches of bananas; however, it is now evening, and she has returned to purchase 2 more bunches. She also visited the supermarket twice yesterday for bananas and is appropriately dressed. Participants will read each vignette and, for each one, will be asked to indicate their answer to the item “You will help her.” The intention to help will be scored using a Likert scale (1=strongly disagree, 2=disagree, 3=agree, and 4=strongly agree). A high score indicates a high intention to help people living with dementia.

##### Demographic Data

Demographic information will be collected, including participants’ age, sex, affiliation, relationship to people living with dementia, experience in assisting and caring for elderly individuals (including people living with dementia), and history of attending dementia-related lectures, whether through a formal course or a dementia-focused organization.

### Data Analysis

Statistical analyses will be performed as follows: initially, descriptive analyses will be conducted to outline the characteristics of the participants. Subsequently, we will evaluate changes in total and subscale scores for attitudes toward people living with dementia, intention to help people living with dementia, knowledge of dementia, and stigma associated with people living with dementia before and after the program using paired *t* tests and the Wilcoxon rank sum test. To determine the effectiveness of the program, we will calculate effect sizes using Cohen *d*, classifying differences between pre- and postprogram scores as small (*d*=0.2), medium (*d*=0.5), or large (*d*=0.8). We will set the threshold for statistical significance at *P*<.05 (2-tailed). The statistical analysis will be conducted using IBM SPSS Statistics (version 26.0).

### Ethical Considerations

The study protocol of this study has been reviewed and approved by the ethical committee of the Faculty of Nursing at Universitas Airlangga (2377-KEPK).

## Results

Data will be collected from November 2023 to March 2024. Data analysis will be completed by May 2024, and the final results will be completed by July 2024. The impact of gaming-based dementia educational programs on enhancing attitudes toward people living with dementia remains unknown. The study findings will be disseminated through publication in a peer-reviewed journal.

## Discussion

### Principal Findings

Our study results will highlight the impact of gaming-based dementia educational programs on enhancing attitudes toward people living with dementia. The model by Lane hypothesized that knowledge influences attitudes and, indirectly, a helping behavior [26]. We will explore the relationships among these variables in our study findings.

The game-based educational program focuses on real-life situations for helping older adults with dementia. Integrating this program with other educational initiatives may amplify its beneficial effects [22,41]. We hypothesize that the results of our study will support the development of positive attitudes and understanding toward people living with dementia. This is crucial for providing effective, person-centered care that improves the quality of life for people living with dementia and diminishes the stigma linked to their condition.

### Limitations

The limitations of the study include its single-arm pre-post design, which impedes the establishment of causation due to the lack of a control group. Without a control group, attributing the observed outcomes exclusively to the gaming-based intervention is challenging, as other variables may influence the results. Furthermore, the impracticality of setting up a control group, owing to the limited number of facilitators, further complicates the isolation of the intervention’s effects. Addressing these limitations is essential for future research to ensure more reliable conclusions regarding the effectiveness of the interventions.

Furthermore, it is important to note that the study’s exclusive emphasis on health care students may restrict its generalizability, and the absence of objective behavioral assessments poses a challenge in evaluating actual changes in behavior. Therefore, to enhance the reliability and applicability of the findings, future research should address these limitations by considering diverse populations, using more robust study designs, and incorporating objective measures.

### Conclusions

To the best of our knowledge, this study represents the inaugural gaming education program among health care students at Indonesian universities, specifically targeting dementia education. This program shows considerable potential as a method to enhance attitudes toward people living with dementia globally, offering a novel approach for the community-based support of people living with dementia.

## Appendix

Multimedia Appendix 1 Overview of The Gaming Educational Program.

Multimedia Appendix 2 Peer-review report by the Research and Community Service Institute, Universitas Airlangga (Indonesia).

## Abbreviations

FSA: Fraboni’s Scale of Ageism
PI: principal investigator

## Data Availability

The data from this study will be stored by the corresponding author and will not be published online.
