# Supplementary material for: Education Program for Enhancing Health Care Students’ Attitudes Toward People Living With Dementia: Protocol for a Single-Arm Pre-Post Study
Source: JMIR Res Protoc. 2024 Sep 18;13:e62654. doi: 10.2196/62654 (PMC11447417; doi:10.2196/62654)
Supplement: Multimedia Appendix 1 [file resprot_v13i1e62654_app1.pdf]

Table 1. Overview of the gaming educational program

The content of the education program	Time
**1) Introduction of the study**<br>Principal investigator explained the overall of study and content of inform	5 min
**2) Pretest**<br>Participants will receive a QR code providing access to information regarding Informed Consent and the online questionnaire.	10 min
**3) Short film (Undesirable scenario)**<br>Observing a narrative featuring a woman people living with dementia (PLWD), participants reflect on her background and the reactions of those around her.	10 min
**4) Lecture**<br>A lecture consists of dementia, treatment of PLWD, person-centered care, and social support. The lecture included questions and answers.	25 min
**5) N-impro gaming**<br>In the gaming setting, students will participate as a group alongside a facilitator. The game is structured with components such as “situation cards,” “answer cards,” and “point cards.” All groups will be provided with identical sets of situation cards.	20 min
**6) Short film (Desirable scenario)**<br>Participants identify differences in the emotional experiences of PLWD and the reactions of individuals in their immediate surroundings between the two given scenarios.	10 min
**7) Posttest**<br>Participants will be issued a QR code to access the online posttest. Additionally, there will be a designated space for participants to provide free-form comments expressing their opinions and feedback on the gaming education program.	10 min
