# Supplementary material for: Education Program for Enhancing Health Care Students’ Attitudes Toward People Living With Dementia: Protocol for a Single-Arm Pre-Post Study
Source: JMIR Res Protoc. 2024 Sep 18;13:e62654. doi: 10.2196/62654 (PMC11447417; doi:10.2196/62654)
Supplement: Multimedia Appendix 2 [file resprot_v13i1e62654_app2.pdf]

KEMENTERIAN PENDIDIKAN, KEBUDAYAAN, RISET, DAN TEKNOLOGI

UNIVERSITAS AIRLANGGA

LEMBAGA PENELITIAN DAN PENGABDIAN MASYARAKAT

Kampus C Mulyorejo Surabaya 60115 - Telp. (031) 5995247 Fax. (0315923584)
laman : http://lppm.unair.ac.id; e-mail : penelitian@lppm.unair.ac.id, pengmas@lppm.unair.ac.id

Number : 46/UN3.LPPM/PT.01.02/2024 January 15th 2024
Subject : Funding Confirmation

To whom it my concern
Dear Sir/Madam,

This letter is to confirm that a scoping review project titled **"Dementia Educational Program for Enhancing Healthcare Students' Attitudes Toward People Living with Dementia: A Single-Arm Pre-Post Study Protocol"** received research grant by Research and Community Service Institute, Universitas Airlangga for the year 2023-2024 (Number 1701/UN3.LPPM/PT.01.03/2023) awarded to Dianis Wulan Sari, S.Kep., Ns., M.HS., Ph.D. This project is peer-reviewed and receives funding of IDR - (approximately USD).

Should you need further information, please do not hesitate to contact the Institute for Research and Community Services.

Best Regards,
Head of Institute Research and Community Service,
Universitas Airlangga

Prof. Dr. Gadis Meinar Sari, dr., M.Kes.
NIP 196605041996032001

Translation of the reviewer's comments

Reviewer 1:

The research addresses the theme related to improving the quality of healthcare workers through educational programs related to understanding people with dementia. The problem statement is described clearly and sharply. There is a research roadmap that is quite clearly illustrated, and information can be gleaned from the publication outputs reported since 2019. The proposer has a substantial track record in terms of research outputs, particularly reputable international publications. There are 5 papers as first author/corresponding author. There is a research collaboration partner from The University of Tokyo.

Suggestion: The protocol related to the questionnaire translation process should be explained in more detail.

Reviewer 2:

The researcher's track record is sufficient, with 10 Scopus publications, an h-index of 4, 69 collaborators, and a SINTA score of 261.

The research substance is good.

Partner: Appropriate from The University of Tokyo (ranked 28) with an h-index of 12.

The research involves students.

Suggestion: SDGs keywords should be included in the research summary, a research flowchart should be added, the roadmap should be made according to the guidelines, and the bibliography should be written according to standard guidelines.

KEMENTRIAN PENDIDIKAN, KEBUDAYAAN, RISET, DAN TEKNOLOGI
UNIVERSITAS AIRLANGGA
LEMBAGA PENELITIAN DAN PENGABDIAN MASYARAKAT
Kampus C Mulyorejo Surabaya 60115 Telp. (031) 5995247 Fax (031) 5923584
laman : https://lppm.unair.ac.id, e-mail : penelitian@lppm.unair.ac.id, pengmas@lppm.unair.ac.id

The reviewer's comments in Indonesian

No	Ketua	Judul Penelitian	Fakultas	Skema	Catatan Reviewer 1	Catatan Reviewer 2
1	Dianis Wulan Sari	Education Program for Enhancing Attitude Toward People Living with Dementia among Nursing Students	Keperawatan	International Research Collaboration on Top #100	Penelitian mengangkat tema terkait peningkatan kualitas tenaga kesehatan melalui program edukasi terkait bagaimana orang demensia. Rumusan permasalahan diuraikan secara jelas dan tajam. Ada roadmap penelitian yang tergambar cukup jelas dan bisa ditangkap informasi dari luaran publikasi yang dilaporkan sejak tahun 2019. pengusul memiliki rekam jejak yang cukup dalam hal luaran penelitian, terutama publikasi internasional bereputasi. Ada 5 paper sebagai first author/ corresponding author. Ada mitra kolaborasi penelitian dari The University of Tokyo. Saran: protokol terkait proses penerjemahan kuesioner dijelaskan lebih rinci lagi.	Rekam jejak peneliti cukup, mempunyai 10 publikasi scopus, h-index = 4, kolaborator 69, score sinta 261. Substansi penelitian baik. Mitra: sesuai dari The University of Tokyo (peringkat 28) dengan H-Index = 12. Penelitian melibatkan mahasiswa. Saran: kata kunci SDGs : dimasukkan dalam ringkasan penelitian, ditambahkan bagan alur penelitian, ditambahkan bagan alur penelitian, roadmap dibuat sesuai panduan, penulisan daftar pustakan dibuat sesuai panduan yang standart.
